# Supplementary material for: ALK ligand ALKAL2 potentiates MYCN‐driven neuroblastoma in the absence of ALK mutation
Source: EMBO J. 2021 Jan 7;40(3):e105784. doi: 10.15252/embj.2020105784 (PMC7849294; doi:10.15252/embj.2020105784)
Supplement: Supplementary file 1 — Appendix [file EMBJ-40-e105784-s001.pdf]

Appendix, Borenäs et al., 2020

Table of contents:

Appendix Figure S1................. 2

Appendix Figure S2...3

Appendix Figure S3...4

Appendix Figure S4...5

Appendix Figure S5...6

Appendix Figure S6...7

Appendix Figure Legends...8

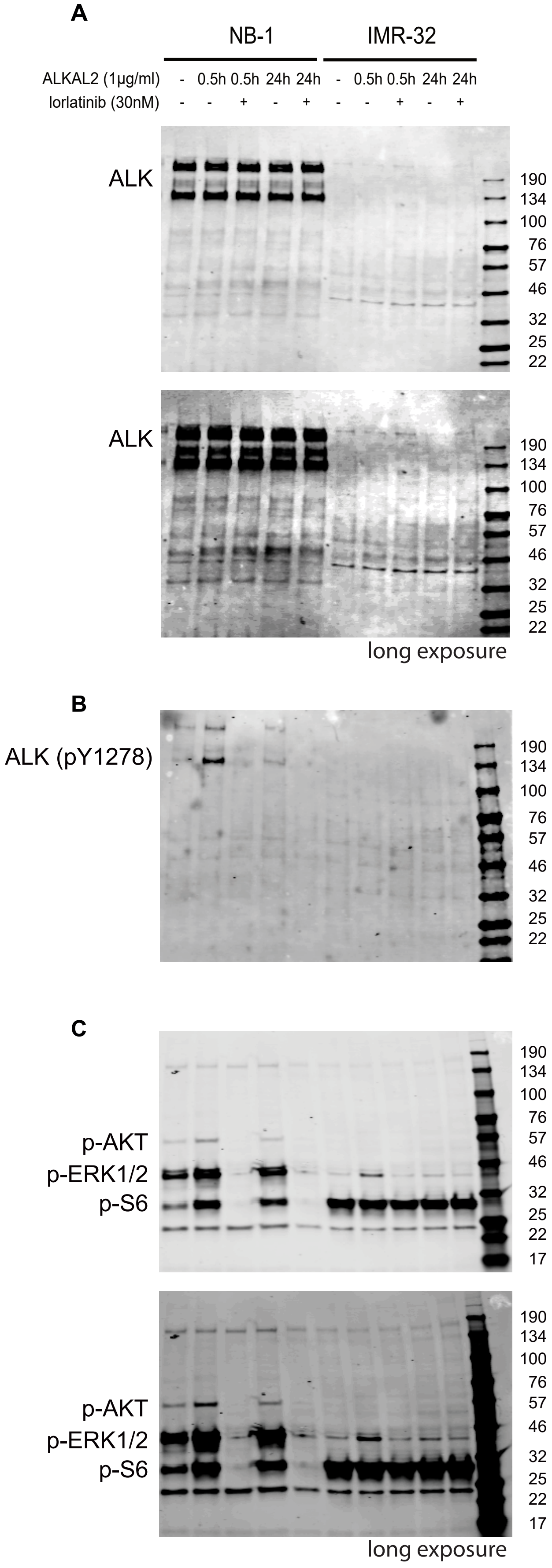

Appendix Figure S1, Borenäs et al., 2020

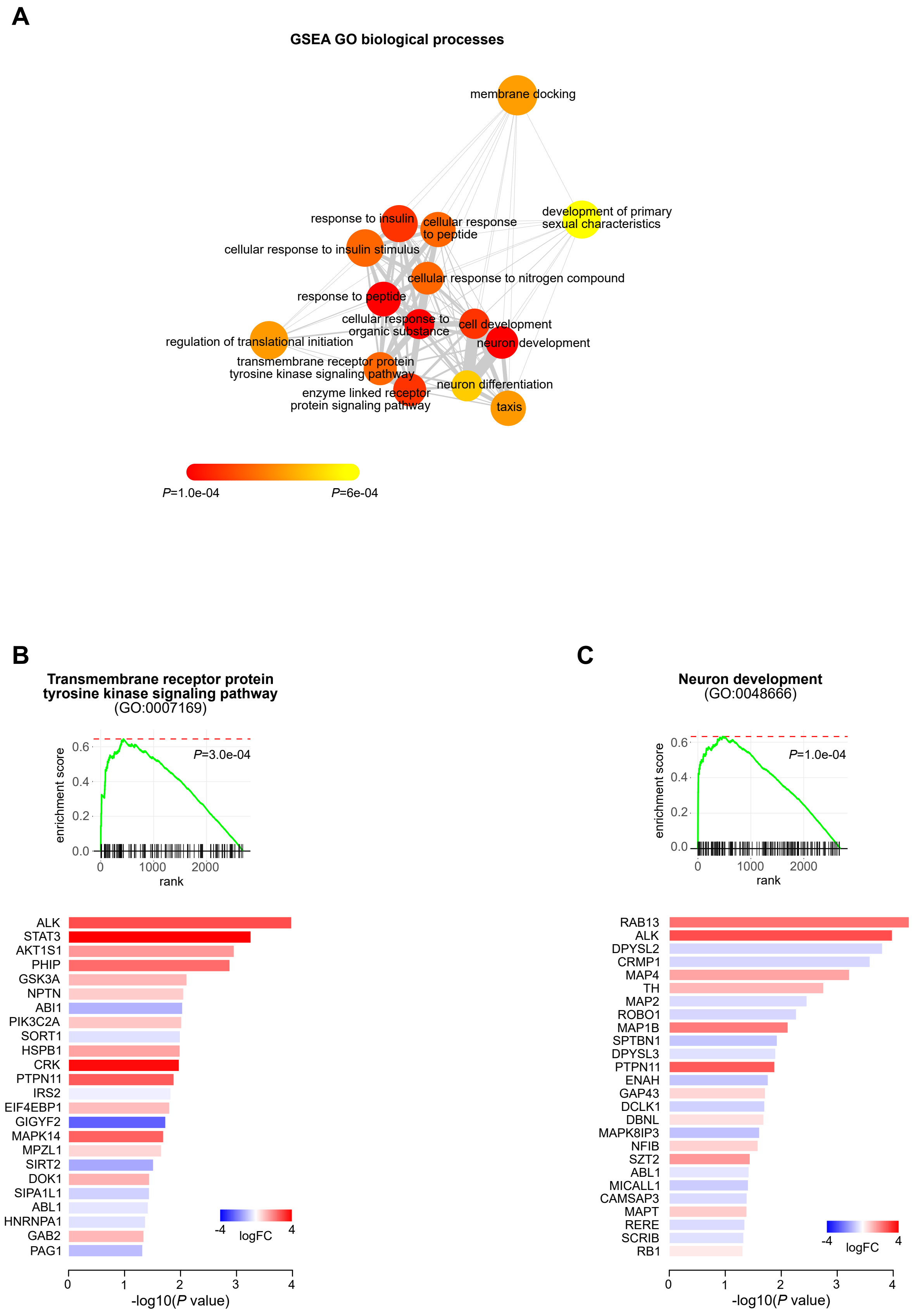

Appendix Figure S2, Borenäs et al., 2020

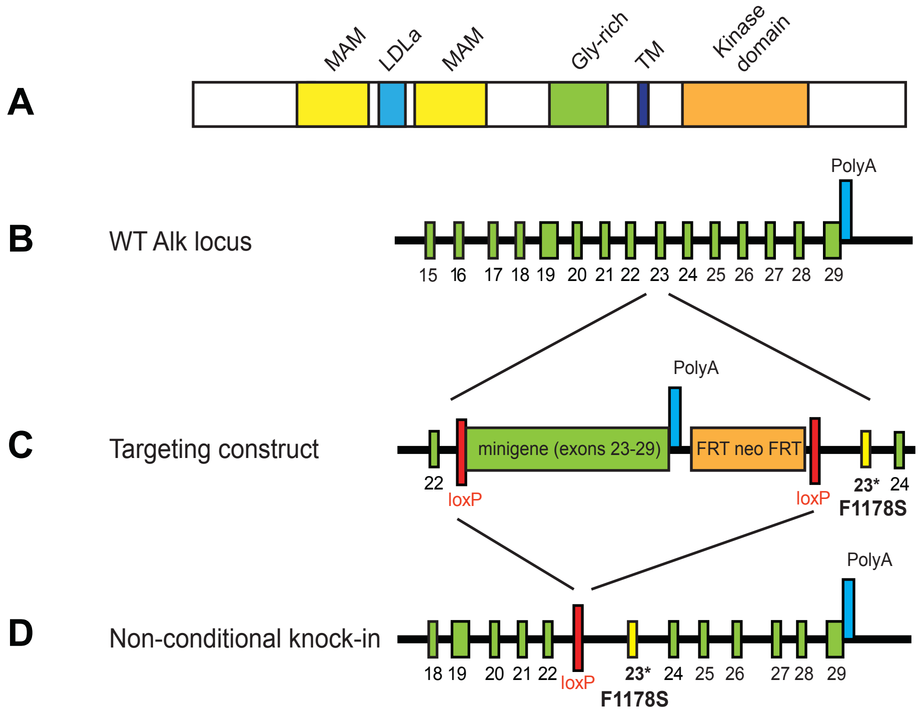

Appendix Figure S3, Borenäs et al., 2020

A

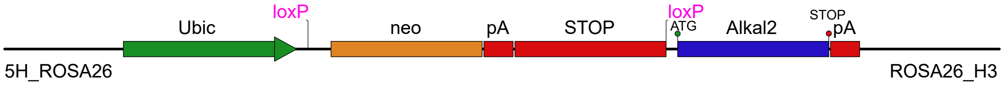

GGCCTCCGCGCCGGGTTTTGGCGCCTCCCGCGGGCGCCCCCCTCCTCACGGCGAGCGCTGCCACGTCAGACGAAGGGCGCAGCGAGCGTCCTGATCCTTCCGCCCGGACGCTCAGGACAGCGGCCCGCTGCTCATAAGACTCG
GCCTTAGAACCCCAGTATCAGCAGAAGGACATTTTAGGACGGGACTTGGGTGACTCTAGGGCACTGGTTTTCTTTCCAGAGAGCGGAACAGGCGAGGAAAAGTAGTCCCTTCTCGGCGATTCTGCGGAGGGATCTCCGTGGGGCGG
TGAACGCCGATGATTATATAAGGACGCGCCGGGTGTGGCACAGCTAGTTCCGTCGCAGCCGGGATTTGGGTCGCGGTTCTTGTTTGTGGATCGCTGTGATCGTCACTTGGTGAGTAGCGGGCTGCTGGGCTGGCCGGGGCTTTCGT
GGCCGCCGGGCCGCTCGGTGGGACGGAAGCGTGTGGAGAGACCGCCAAGGGCTGTAGTCTGGGTCCGCGAGCAAGGTTGCCCTGAACTGGGGGTTGGGGGGAGCGCAGCAAAATGGCGGCTGTTCCCGAGTCTTGAATGGAAG
ACGCTTGTGAGGCGGGCTGTGAGGTCGTTGAAACAAGGTGGGGGGCATGGTGGGCGGCAAGAACCCAAGGTCTTGAGGCCTTCGCTAATGCGGGAAAGCTCTTATTCGGGTGAGATGGGCTGGGGCACCATCTGGGGACCCTGA
CGTGAAGTTTGTCACTGACTGGAGAACTCGGTTTGTCGTCTGTTGCGGGGGCGGCAGTTATGGCGGTGCCGTTGGGCAGTGCACCCGTACCTTTGGGAGCGCGCGCCCTCGTCGTGTCGTGACGTCACCCGTTCTGTTGGCTTATA
ATGCAGGGTGGGGCCACCTGCCGGTAGGTGTGCGGTAGGCTTTTCTCCGTCGCAGGACGCAGGGTTCGGGCCTAGGGTAGGCTCTCCTGAATCGACAGGCGCCGGACCTCTGGTGAGGGGAGGGATAAGTGAGGCGTCAGTTTC
TTTGGTCGGTTTTATGTACCTATCTTCTTAAGTAGCTGAAGCTCCGGTTTTGAACTATGCGCTCGGGGTTGGCGAGTGTGTTTTGTGAAGTTTTTTAGGCACCTTTTGAAATGTAATCATTTGGGTCAATATGTAATTTTCAGTGTTAGACTA
GTAAATTGTCCGCTAAATTCTGGCCGTTTTTGGCTTTTTTGTTAGACAATTACATCATAACTTCGTATAGCATACATTATACGAAGTTATTTAAGATCTAAACCGCCACCATGGGATCGGCCATTGAACAAGATGGATTGCACGCAGGTTCTC
CGGCCGCTTGGGTGGAGAGGCTATTCGGCTATGACTGGGCACAACAGACAATCGGCTGCTCTGATGCCGCCGTGTTCCGGCTGTCAGCGCAGGGGCGCCCGGTTCTTTTTGTCAAGACCGACCTGTCCGGTGCCCTGAATGAACT
GCAGGACGAGGCAGCGCGGCTATCGTGGCTGGCCACGACGGGCGTTCCTTGCGCAGCTGTGCTCGACGTTGTCACTGAAGCGGGAAGGGACTGGCTGCTATTGGGCGAAGTGCCGGGGCAGGATCTCCTGTCATCTCACCTTGC
TCCTGCCGAGAAAGTATCCATCATGGCTGATGCAATGCGGCGGCTGCATACGCTTGATCCGGCTACCTGCCCATTCGACCACCAAGCGAAACATCGCATCGAGCGAGCACGTACTCGGATGGAAGCCGGTCTTGTCGATCAGGATGAT
CTGGACGAAGAGCATCAGGGGCTCGCGCCAGCCGAACTGTTCGCCAGGCTCAAGGCGCGCATGCCCGACGGCGATGATCTCGTCGTGACCCATGGCGATGCCTGCTTGCCGAATATCATGGTGGAAAATGGCCGCTTTTCTGGATT
CATCGACTGTGGCCGGCTGGGTGTGGCGGACCGCTATCAGGACATAGCGTTGGCTACCCGTGATATTGCTGAAGAGCTTGGCGGCGAATGGGCTGACCGCTTCCTCGTGCTTTACGGTATCGCCGCTCCCGATTCGCAGCGCATCG
CCTTCTATCGCCTTCTTGACGAGTTCTTCTGAGGGGATCAATTCTCTAGCTAGAGCTCGCTGATCAGCCTCGACTGTGCCTTCTAGTTGCCAGCCATCTGTTGTTTGCCCCTCCCCCGTGCCTTCCTTGACCCTGGAAGGTGCCACTCC
CACTGTCCTTTCCTAATAAAATGAGGAAATTGCATCGCATTGTCTGAGTAGGTGTCATTCTATTCTGGGGGGTGGGGTGGGGCAGGACAGCAAGGGGGAGGATTGGGAAGACAATAGCAGGCATGCTGGGGATGCGGTGGGCTCTAT
GGCTTCTGAGGCGGAAAGAACCATCTAGCTACAAACTCCTCGGGGACACCAAATATGGCGATCTCGGCCTTTTCGTTTCTTGGAGCTGGGACATGTTTGCCATCGATCCATCTACCACCAGAACGGCCGTTAGATCTGCTGCCACCGTT
GTTTCCACCGAAGAAACCACCGTTGCCGTAACCACCACGACGGTTGTTGCTAAAGAAGCTGCCACCGCCACGGCCACCGTTGTAGCCGCCGTTGTTGTTATTGTAGCTGCTACTGTTATTTCTGGCACTTCTTGGTTTTCCTCTTAAGT
GAGGAGGAACATAACCATTCTCGTTGTTGTCGTTGATGCTTAAATTTTGCACTTGTTCGCTCAGTTCAGCCATAATATGAAATGCTTTTCTTGTTGTTCTTACGGAATACCACTTGCCACCTATCACCACAACTAACTTTTTCCCGTTCCTCC
ATCTCTTTTATATTTTTTTTCTCGAGGGATCTTTGTGAAGGAACCTTACTTCTGTGGTGTGACATAATTGGACAAACTACCTACAGAGGTTTAAAGCTCTAAGGTAAATATAAAATTTTTAAGTGTATAATGTGTTAAACTACTGATTCTAATTGT
TTGTGTATTTTAGATTCCAACCTATGGAACTGATGAATGGGAGCAGTGGTGGAATGCCTTTAATGAGGAAAACCTGTTTTGCTCAGAAGAAATGCCATCTAGTGATGATGAGGCTACTGCTGACTCTCAACATTCTACTCCTCCAAAAAAG
AAGAGAAAGGTAGAAGACCCCAAGGACTTTCCTTCAGAATTGCTAAGTTTTTTGAGTCATGCTGTGTTTAGTAATAGAACTCTTGCTTGCTTTGCTATTTACACCACAAAGGAAAAAGCTGCACTGCTATACAAGAAAATTATGGAAAAATAT
TCTGTAACCTTTATAAGTAGGCATAACAGTTATAATCATAACATACTGTTTTTTCTTACTCCACACAGGCATAGAGTGTCTGCTATTAATAACTATGCTCAAAAATTGTGTACCTTTAGCTTTTTAATTTGTAAAGGGGTTAATAAGGAATATTTGA
TGTATAGTGCCTTGACTAGAGATCATAATCAGCCATACCACATTTGTAGAGGTTTTACTTGCTTTAAAAAACCTCCCACACCTCCCCCTGAACCTGAAACATAAAATGAATGCAATTGTTGTTGTTAACTTGTTTATTGCAGCTTATAATGGTT
ACAAATAAAGCAATAGCATCACAAATTTCACAAATAAAGCATTTTTTTCACTGCATTCTAGTTGTGGTTTGTCCAAACTCATCAATGTATCTTATCATGTCTGGATCTGACATGGTAAGTAAGCTTGGGCTGCAGGTCGAGGGACCTAATAAC
TTCGTATAGCATACATTATACGAAGTTATTTAAGATCTAAACCGCCACCATGCGAGTCTCAGGTAGACCTATGCTGCTGGCCCTCCTGTTGCTGCTTAGCACCGTAGGGGACCGGGGTAGAGCACAGAGCCGGGGCCCAGCCGACCGG
CAAACACTCTTGAGACTTCTGGTGGAACTTGTTCAGGAGCTGAAGAAGTTTCATATTGGCGACTCAAAAAGGCTGCAGCTTCTGGGGGAATCTGACTTTGCTCTCGGTCGACGGGAAGCTACCGACTATGGAGCCGATCAGGAGGAG
CAGCGCGTGGAAATCGTACCTAGAGATCTTAGAATGAAGGACAAGTTCCTGAAGCACCTTACGGGCCCATTGTACTTCTCTCCTAAGTGTAGCAAACATTTCCACAGACTCTATCACAACACAAGAGATTGTACGATCCCTGCTTACTACA
AGCGGTGTGCCAGGCTGTTGACTCGGCTGGCTGTCAGCCCCATGTGTATGGAACGGTAGGGCGCGCCCTGTGCCTTCTAGTTGCCAGCCATCTGTTGTTTGCCCCTCCCCCGTGCCTTCCTTGACCCTGGAAGGTGCCACTCCCAC
TGTCCTTTCCTAATAAAATGAGGAAATTGCATCGCATTGTCTGAGTAGGTGTCATTCTATTCTGGGGGGTGGGGTGGGGCAGGACAGCAAGGGGGAGGATTGGGAAGACAATAGCAGGCATGCTGGGGATGCGGTGGGCTCTATGG

B

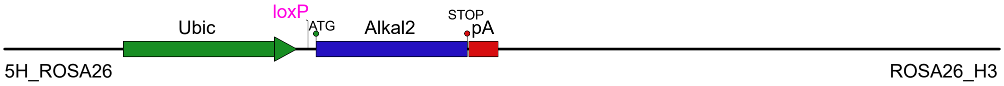

GGCCTCCGCGCCGGGTTTTGGCGCCTCCCGCGGGCGCCCCCCTCCTCACGGCGAGCGCTGCCACGTCAGACGAAGGGCGCAGCGAGCGTCCTGATCCTTCCGCCCGGACGCTCAGGACAGCGGCCCGCTGCTCATAAGACTCG
GCCTTAGAACCCCAGTATCAGCAGAAGGACATTTTAGGACGGGACTTGGGTGACTCTAGGGCACTGGTTTTCTTTCCAGAGAGCGGAACAGGCGAGGAAAAGTAGTCCCTTCTCGGCGATTCTGCGGAGGGATCTCCGTGGGGCGG
TGAACGCCGATGATTATATAAGGACGCGCCGGGTGTGGCACAGCTAGTTCCGTCGCAGCCGGGATTTGGGTCGCGGTTCTTGTTTGTGGATCGCTGTGATCGTCACTTGGTGAGTAGCGGGCTGCTGGGCTGGCCGGGGCTTTCGT
GGCCGCCGGGCCGCTCGGTGGGACGGAAGCGTGTGGAGAGACCGCCAAGGGCTGTAGTCTGGGTCCGCGAGCAAGGTTGCCCTGAACTGGGGGTTGGGGGGAGCGCAGCAAAATGGCGGCTGTTCCCGAGTCTTGAATGGAAG
ACGCTTGTGAGGCGGGCTGTGAGGTCGTTGAAACAAGGTGGGGGGCATGGTGGGCGGCAAGAACCCAAGGTCTTGAGGCCTTCGCTAATGCGGGAAAGCTCTTATTCGGGTGAGATGGGCTGGGGCACCATCTGGGGACCCTGA
CGTGAAGTTTGTCACTGACTGGAGAACTCGGTTTGTCGTCTGTTGCGGGGGCGGCAGTTATGGCGGTGCCGTTGGGCAGTGCACCCGTACCTTTGGGAGCGCGCGCCCTCGTCGTGTCGTGACGTCACCCGTTCTGTTGGCTTATA
ATGCAGGGTGGGGCCACCTGCCGGTAGGTGTGCGGTAGGCTTTTCTCCGTCGCAGGACGCAGGGTTCGGGCCTAGGGTAGGCTCTCCTGAATCGACAGGCGCCGGACCTCTGGTGAGGGGAGGGATAAGTGAGGCGTCAGTTTC
TTTGGTCGGTTTTATGTACCTATCTTCTTAAGTAGCTGAAGCTCCGGTTTTGAACTATGCGCTCGGGGTTGGCGAGTGTGTTTTGTGAAGTTTTTTAGGCACCTTTTGAAATGTAATCATTTGGGTCAATATGTAATTTTCAGTGTTAGACTA
GTAAATTGTCCGCTAAATTCTGGCCGTTTTTGGCTTTTTTGTTAGACAATTACATCATAACTTCGTATAGCATACATTATACGAAGTTATTTAAGATCTAAACCGCCACCATGCGAGTCTCAGGTAGACCTATGCTGCTGGCCCTCCTGTTGC
TGCTTAGCACCGTAGGGGACCGGGGTAGAGCACAGAGCCGGGGCCCAGCCGACCGGCAAACACTCTTGAGACTTCTGGTGGAACTTGTTCAGGAGCTGAAGAAGTTTCATATTGGCGACTCAAAAAGGCTGCAGCTTCTGGGGGAA
TCTGACTTTGCTCTCGGTCGACGGGAAGCTACCGACTATGGAGCCGATCAGGAGGAGCAGCGCGTGGAAATCGTACCTAGAGATCTTAGAATGAAGGACAAGTTCCTGAAGCACCTTACGGGCCCATTGTACTTCTCTCCTAAGTGTA
GCAAACATTTCCACAGACTCTATCACAACACAAGAGATTGTACGATCCCTGCTTACTACAAGCGGTGTGCCAGGCTGTTGACTCGGCTGGCTGTCAGCCCCATGTGTATGGAACGGTAGGGCGCGCCCTGTGCCTTCTAGTTGCCAGC
CATCTGTTGTTTGCCCCTCCCCCGTGCCTTCCTTGACCCTGGAAGGTGCCACTCCCACTGTCCTTTCCTAATAAAATGAGGAAATTGCATCGCATTGTCTGAGTAGGTGTCATTCTATTCTGGGGGGTGGGGTGGGGCAGGACAGCAA
GGGGGAGGATTGGGAAGACAATAGCAGGCATGCTGGGGATGCGGTGGGCTCTATGG

Appendix Figure S4, Borenäs et al., 2020

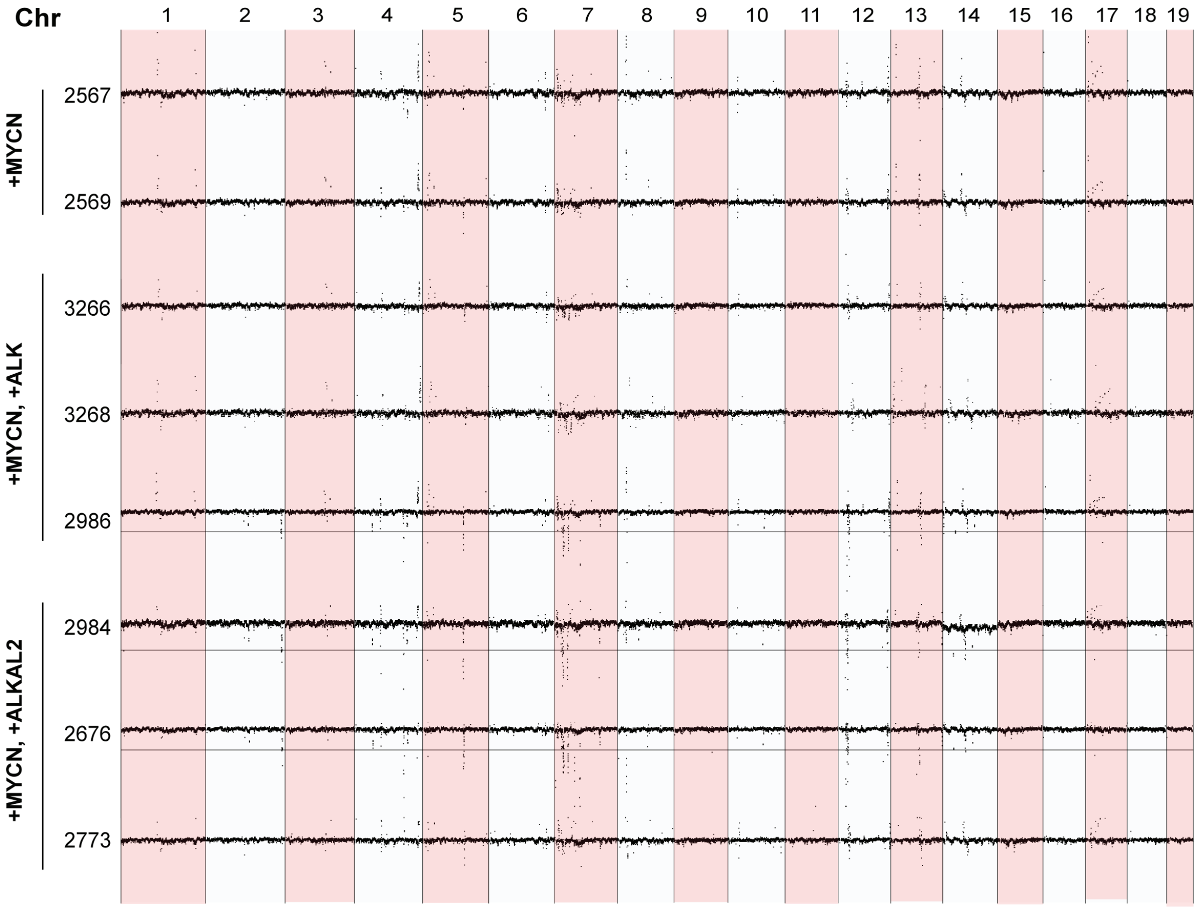

Appendix Figure S5, Borenäs et al., 2020

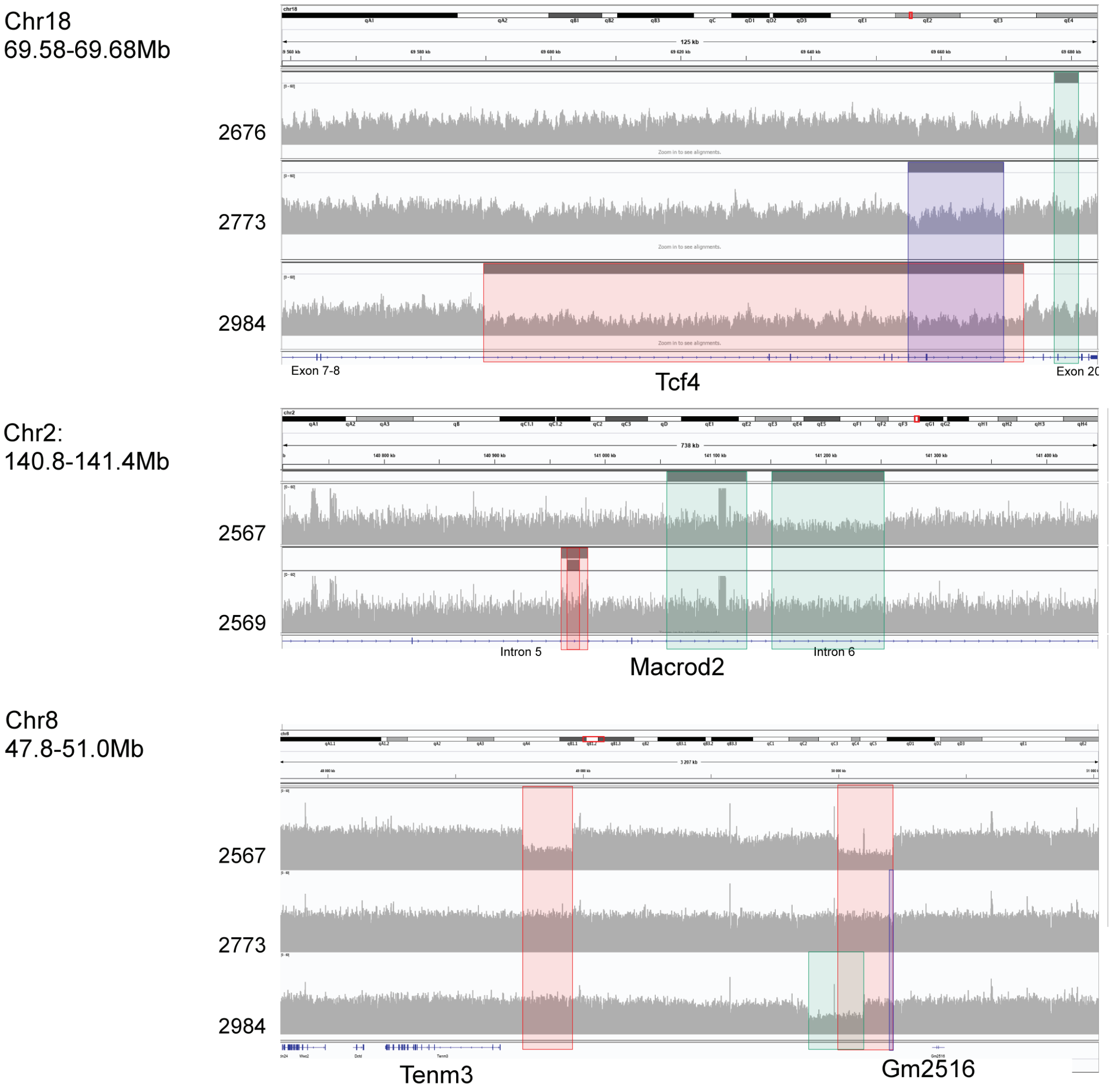

Appendix Figure S6, Borenäs et al., 2020

Appendix Figure S1. ALKAL2 stimulation of NB1 and IMR-32 cells.

A-C. Immunoblotting analysis for total ALK **(A)**, p-ALK **(B)**, p-AKT, p-ERK1/2 and p-S6 **(C)** in NB1 and IMR-32 cells after stimulation with ALK ligand (ALKAL2) for 30 min or 24 h in the presence or absence of the ALK inhibitor, lorlatinib as indicated. Whole cell lysates (20g) were analysed in each lane. Long exposures are shown for **(A)** and **(C)**, due to the very weak signals observed in IMR-32 cells.

Appendix Figure S2. Gene Ontology (GO) GSEA of differentially phosphorylated proteins.

Differential phosphorylation was determined in NB1 cells in response to 1h ALKAL2 stimulation. A ranked GSEA was performed with proteins ranked based on the absolute value of the ROTS statistic (see *Methods*).

A. GSEA network graph with nodes representing the enriched GO biological processes (at 5% FDR). Node sizes correlate to the normalized enrichment scores, node colors indicate *P* values (as in color legend) and edge widths correspond to the number of overlapping genes between the connected nodes.

B-C. Barplots showing all proteins with differentially phosphorylated sites that are involved in the enriched **(B)** GO transmembrane receptor tyrosine kinase signaling pathway (GO:0007169) or **(C)** GO neuron development (GO:0048666). Enrichment running score plots shown on top). Bar colors correspond to logFC values as indicated by color scale.

Appendix Figure S3. *ALK-F1178S* mouse construction.

A. Schematic of the ALK receptor tyrosine kinase protein, depicting the Meprin, A5 protein, and protein tyrosine phosphatase Mu (MAM) domains, Low-density lipoprotein receptor domain class A (LDLa), glycine-rich region (Gly-rich), transmembrane region (TM) and kinase domain.

B-D. A targeting construct containing a minigene of *Alk* exons 23-29 **(C)** was inserted into the endogenous *Alk* locus **(B)** by means of homologues recombination. The targeting construct contained 3’ and 5’ homology sequences and the loxP flanked minigene *Alk* cDNA corresponding to wild type *Alk* exons 23-29 and a neomycin selection cassette, following by the mutated exon 23 containing residue F1178S (ttc>tcc). Upon homologous recombination, FLP and Cre recombination, the modified *Alk* locus in *Alk-F1178S* animals harbors a mutated exon 23 **(D)**.

Appendix Figure S4. Overview of *Rosa26_Alkal2* transgenic mice.

A. Schematic view of the *Rosa26_Alkal2* transgenic insertion is shown. The corresponding sequence is shown below with color coding of base pairs reflecting the indicated region in the schematic above.

B. A schematic view of the *Rosa26_Alkal2* transgenic insertion after loxP/Cre deletion is shown. The corresponding sequence is shown below with color coding of base pairs reflecting the indicated region in the schematic above.

Appendix Figure S5. Whole genome sequencing of *Th-MYCN*, *Alk-F1178S;Th-MYCN* and *Rosa26_Alkal2;Th-MYCN* tumors. Copy number profiling based on normalized WGS coverage data showed lack of larger segmental aberrations.

Appendix Figure S6. Microdeletions detected by WGS of mouse NB. Smaller focal deletions or gains (37) were detected with an average of 3.8 per sample (range 1-7), with recurrent alterations affecting three different genomic loci. These alterations included deletions of the transcription factor encoding gene *Tcf4* on chromosome 18, as observed in three tumors harvested from three independent *Th-MYCN;Rosa26-Alkal2*-driven mice (#2676, #2773 and #2984). Intronic deletions of Macrod2 were seen in two *Th-MYCN* tumors (#2567 and #2569). In addition, three of the analyzed tumors (#2567, #2773 and #2984) harbored smaller deletions in chromosomal region 8qb1.2 whereof one had two deletions in close proximity of each other. These deletions on chr8 clustered in a region located approximately 1500 kb distal of the transcription start of *Tenm3* and 250 kb proximal to the transcription start of *Gm2516*.
