# Supplementary material for: ALK ligand ALKAL2 potentiates MYCN‐driven neuroblastoma in the absence of ALK mutation
Source: EMBO J. 2021 Jan 7;40(3):e105784. doi: 10.15252/embj.2020105784 (PMC7849294; doi:10.15252/embj.2020105784)

Expanded View Figures

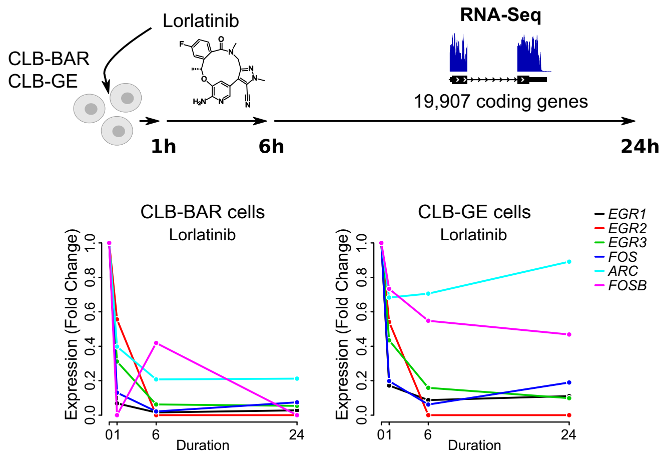

Figure EV1. Inhibition of early response gene expression by lorlatinib in ALK-driven neuroblastoma cells.

Lorlatinib-induced gene expression changes of *ARC*, *EGR1-3*, *FOS* and *FOSB* in NB cell lines that harbor ALK activating mutations. Data obtained from (Van den Eynden *et al*, 2018).

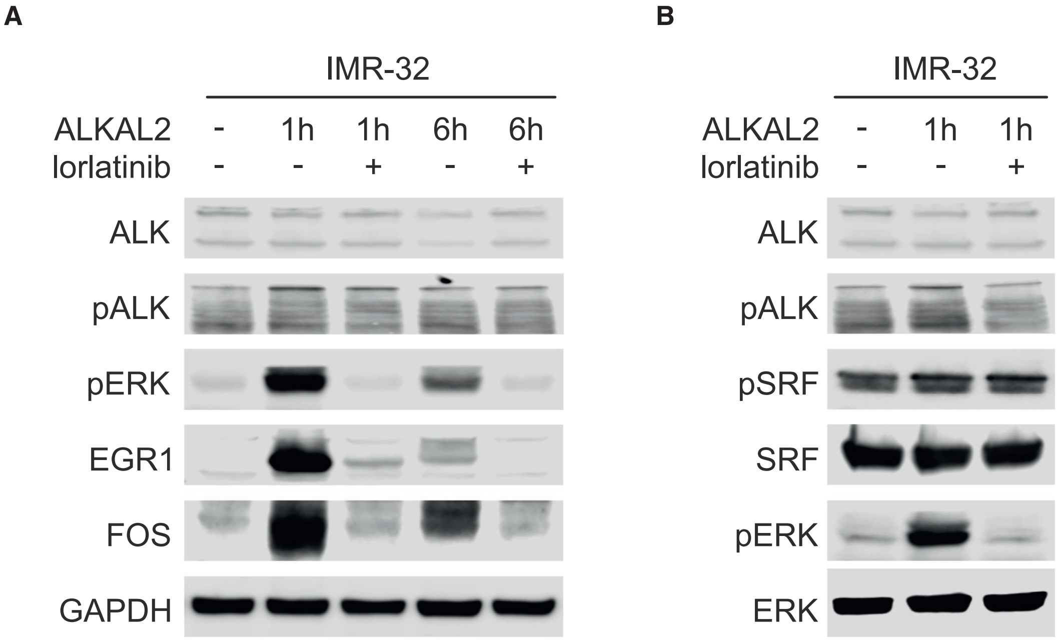

Figure EV2. Immunoblot validation of ALKAL2 induction of EGR1 and FOS at the protein level in IMR32 cells.

A IMR32 cells were treated for 0, 1 and 6 h in the presence and absence of lorlatinib as indicated. Lysates were immunoblotted for EGR1 and FOS, as well as pERK as readout of ALKAL2-induced ALK signalling.

B IMR32 cells were treated for 0 or 1 h in the presence and absence of lorlatinib as indicated. Lysates were immunoblotted for pSRF and SRF, as well as pERK as readout of ALKAL2-induced ALK signalling.

© 2021 The Authors

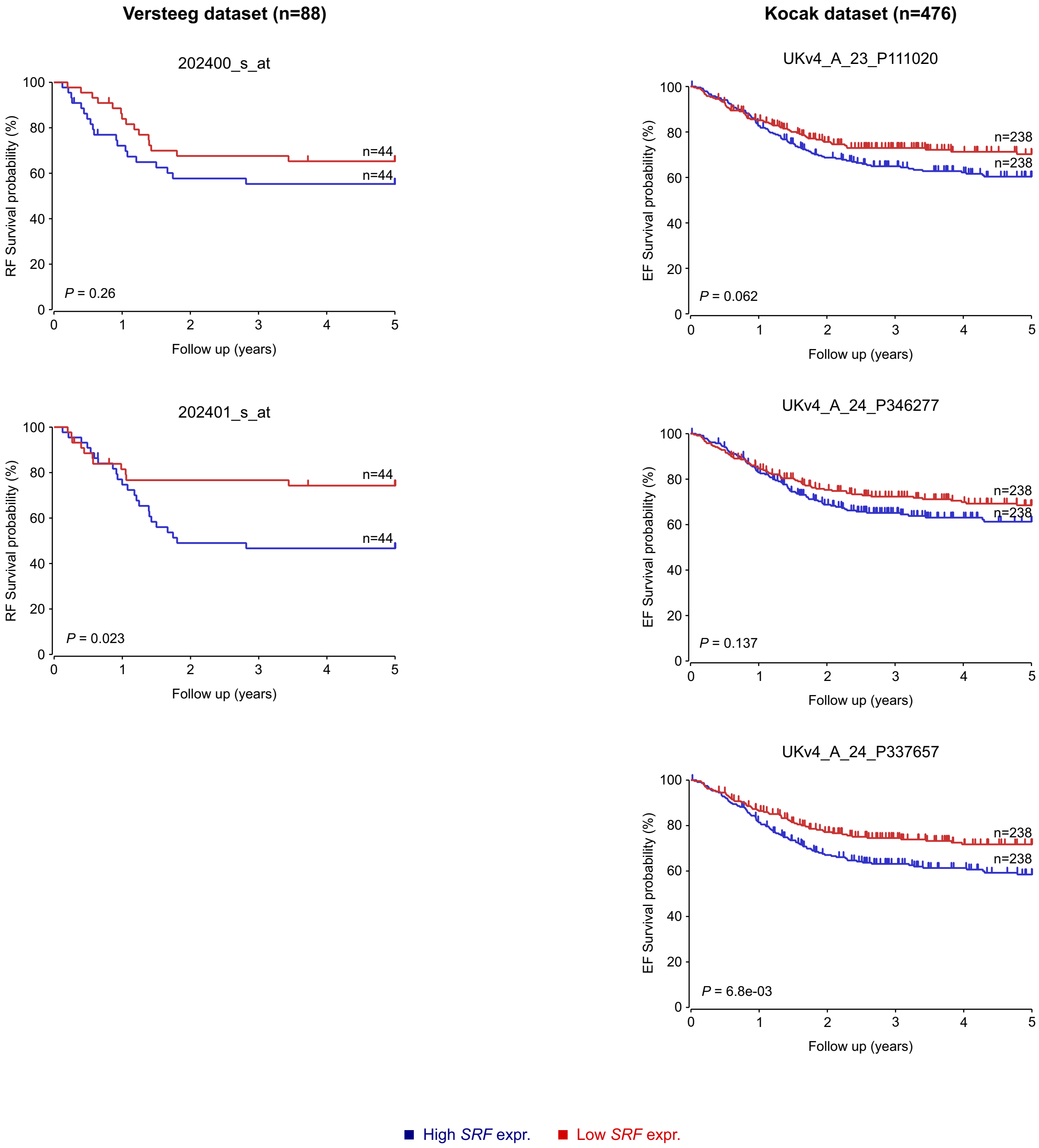

Figure EV3. Kaplan-Meier relapse/event free survival probability curves for *SRF*.

Kaplan-Meier relapse/event free survival probability curves from the NB Versteeg cohort (left panels, 2 different probes) and the Kocak cohort (right panels, 3 different probes), as derived from the R2 platform. Patients with higher SRF expression are highlighted in blue, whereas patients with lower expression are highlighted in red. The log-rank test *P* values are indicated.

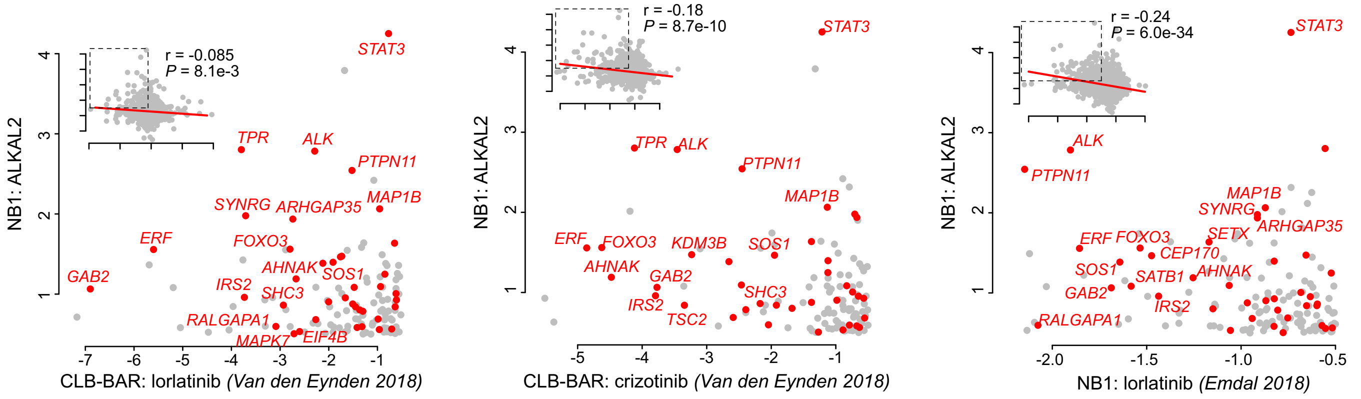

Figure EV4. Correlation between phosphoproteomic responses after ALKAL2 stimulation and ALK inhibition.

Differential phosphorylation was determined in NB1 cells in response to 1h ALKAL2 stimulation. Plots show correlations with $\log_2$FC values determined after inhibiting CLB-BAR or NB1 cell lines with the ALK TKI lorlatinib or crizotinib as indicated. The inset on top left of each plot shows the linear regression line in red with indication of Pearson correlation coefficient and *P* value. Dashed square shows the region that is shown by the main plot. Genes indicated in red show differential phosphorylation ($\log_2$FC below 0.5) in all ALK TKI conditions. When phosphorylation was measured in multiple sites from the same protein, the site with the highest absolute response was used for the analysis. ALK TKI data were obtained from 2 independent studies as indicated (Emdal *et al*, 2018; Van den Eynden *et al*, 2018).

 © 2021 The Authors

A

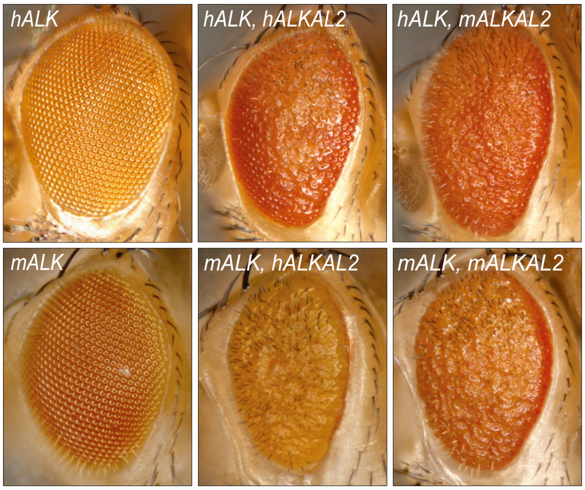

Figure EV5. ALKAL2 activation of ALK is conserved between humans and mice.

A Ectopic expression of human ALK and ALKAL2 in the *Drosophila* eye leads to astrong rough eye phenotype, similar to overexpression of mouse ALK and ALKAL2. Ectopic expression of different mouse/human ALK and ALKAL2 combinations also lead to rough eye phenotypes, indicating ALK activity.

B Endogenous ALK present in NB1 cells is activated upon ALKAL2 stimulation, and responds to lorlatinib treatment. Non-stimulated cells were used as negative controls, cells stimulated with mAb46 were used as positive controls.

C PC12 cells ectopically expressing mouse ALK were stimulated by addition of recombinant human ALKAL2 or mouse ALKAL2. Cells expressing human ALK-Y1278S were used as positive control, cells transfected with the empty pcDNA3 vector were used as negative controls.

B

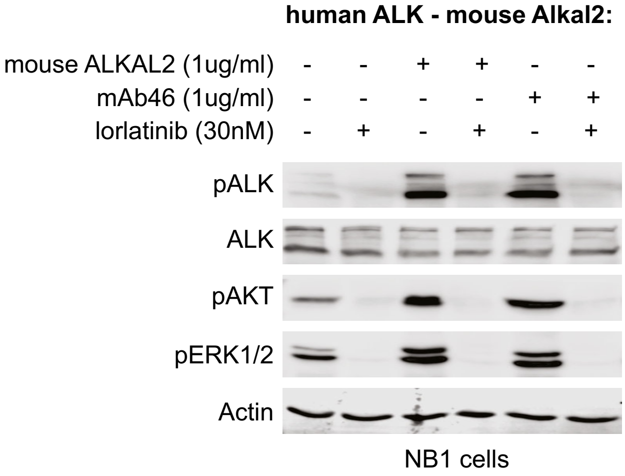

C

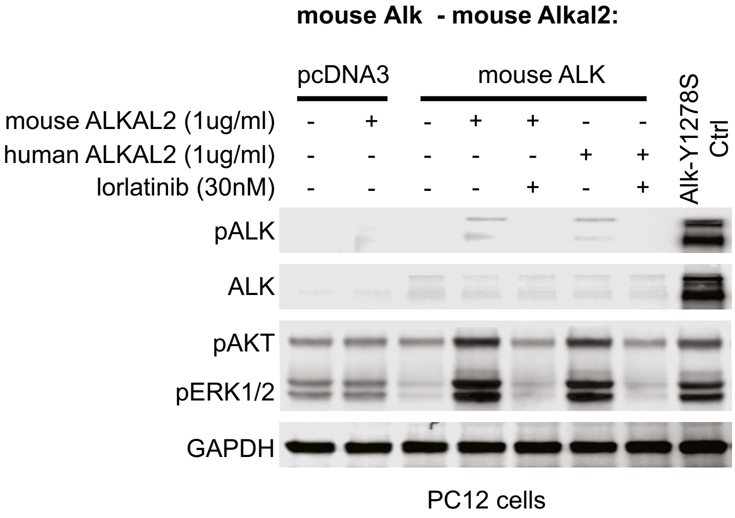

Supplement: Supplementary file 2 — Expanded View Figures PDF [file EMBJ-40-e105784-s002.pdf]
